# Life‐threatening heat‐related illness with severe hyponatremia in an aluminum smelter worker

**DOI:** 10.1002/ajim.23061

**Published:** 2019-10-24

**Authors:** James C. Wesdock, A. Michael Donoghue

**Affiliations:** ^1^ Alcoa Corporation Pittsburgh Pennsylvania; ^2^ Alcoa Alumina Perth Western Australia Australia

**Keywords:** aluminum pot room, encephalopathy, exertional heat stroke, heat‐related illness, hyponatremia

## Abstract

Heat stress is a recognized occupational hazard in aluminum smelter pot rooms. This is the report of an unusual and complex case of heat‐related illness in an aluminum smelter worker. The 34‐year‐old male US worker developed life‐threatening heat‐related illness in August 2018, on his first day back at work after a 7‐day absence. The worker initially presented with bilateral hand then all‐extremity cramping followed some hours later by a generalized seizure and acute mental status changes, including combativeness. Emergency room evaluation identified a serum sodium level of 114 mmol/L. Acute liver and kidney injury ensued along with profound rhabdomyolysis, with peak total creatinine phosphokinase level reaching over 125 000 units/L at 3 days post incident. Initial ventilatory support, careful fluid resuscitation, and electrolyte management were provided. Metabolic encephalopathy resolved. Complications included sepsis. After 5 days in the intensive care unit and eight additional days of inpatient management, observation, and the initiation of rehabilitation, the worker was discharged. Residual effects include polyneuropathy of upper and lower extremities and the postdischarge magnetic resonance imaging finding of a cerebellar lesion. Prevailing considerations in the differential diagnosis included exertional heat stroke and/or exertion‐associated hyponatremia with encephalopathy.

## 1 INTRODUCTION

Heat‐related illness (HRI) is a common but preventable manifestation of exposure to hot and humid environments.^1^, ^2^ Heat stroke is the most severe form of HRI and a life‐threatening condition causing multiorgan system injury. Clinical case definitions of heat stroke typically include core temperature above 40°C (104°F) and central nervous system (CNS) dysfunction as the hallmark features.^3^, ^4^ Altered mental status is the universally regarded clinical feature that distinguishes heat stroke from heat exhaustion and less severe forms of HRI.^5^ Classically described hot, dry skin is variably present and sweating, even profuse, is not uncommon.^5^

Both nonexertional and exertional heat stroke variants exist, with the latter type predominating in occupational settings. A recent analysis of US Occupational Safety and Health Administration (OSHA) data revealed that in 2015 there were 2830 cases of occupational HRI that resulted in at least 1 day of lost work, with 214 hospitalizations and 37 fatalities.^6^ Epidemiologically, the majority (over 70%) of heat‐related fatalities occur during the first week on the job, with nearly half (45%) occurring on the first day on the job or return to duty after an absence of a week or more.^6^

**Figure 1:**
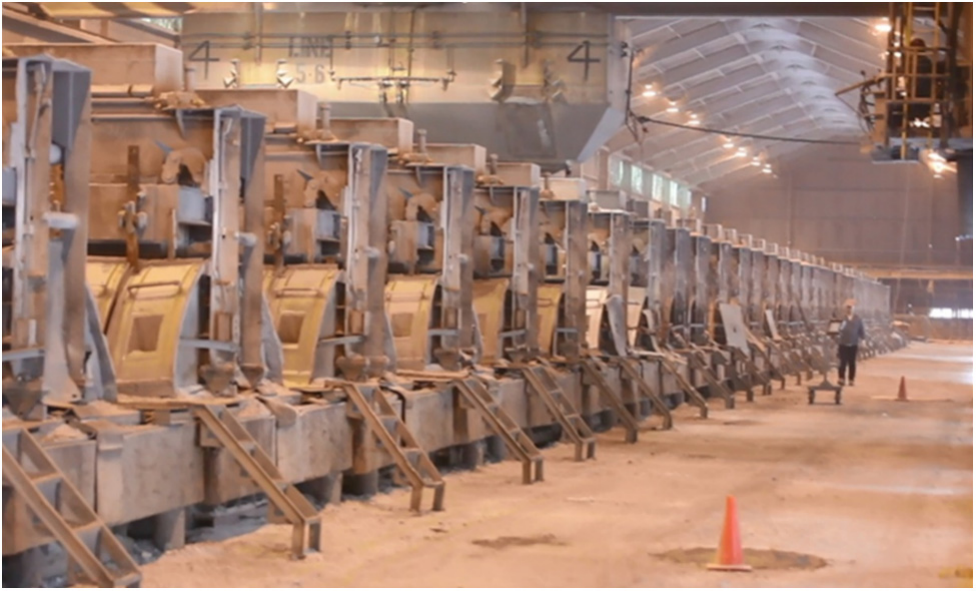
Aluminum smelter pot room showing a series of pots [Color figure can be viewed at wileyonlinelibrary.com]

Aluminum smelter operations entail employee work in proximity to “pots”—aligned in series within a pot room (Figure 1)—in which an electrochemical process reduces alumina ore into molten aluminum metal.^7^, ^8^ The nature of this work subjects employees to significant radiant heat load which, even with mild outdoor ambient thermal conditions, can result in personal heat strain that overwhelms the body's usual compensating mechanisms.^9^, ^10^, ^11^ Consequently, HRI is an acknowledged and not infrequent adverse health outcome of work in pot rooms, although bona fide exertional heat stroke is rare and unreported in the literature to date.^9^, ^10^, ^11^, ^12^

## 2 CASE REPORT

Following a 7‐day scheduled absence from the workplace, a 34‐year old male aluminum smelter pot room process control operator (PCO) returned to work to commence a series of evening shifts from 6.00 PM to 6.00 AM. The first half of the shift involved vigorous and intense pot room control tasks. Following the lunch break (ie, during the second half of the shift), the worker was assigned to other routine, though less demanding, tasks with less heat stress potential.

**Figure 2:**
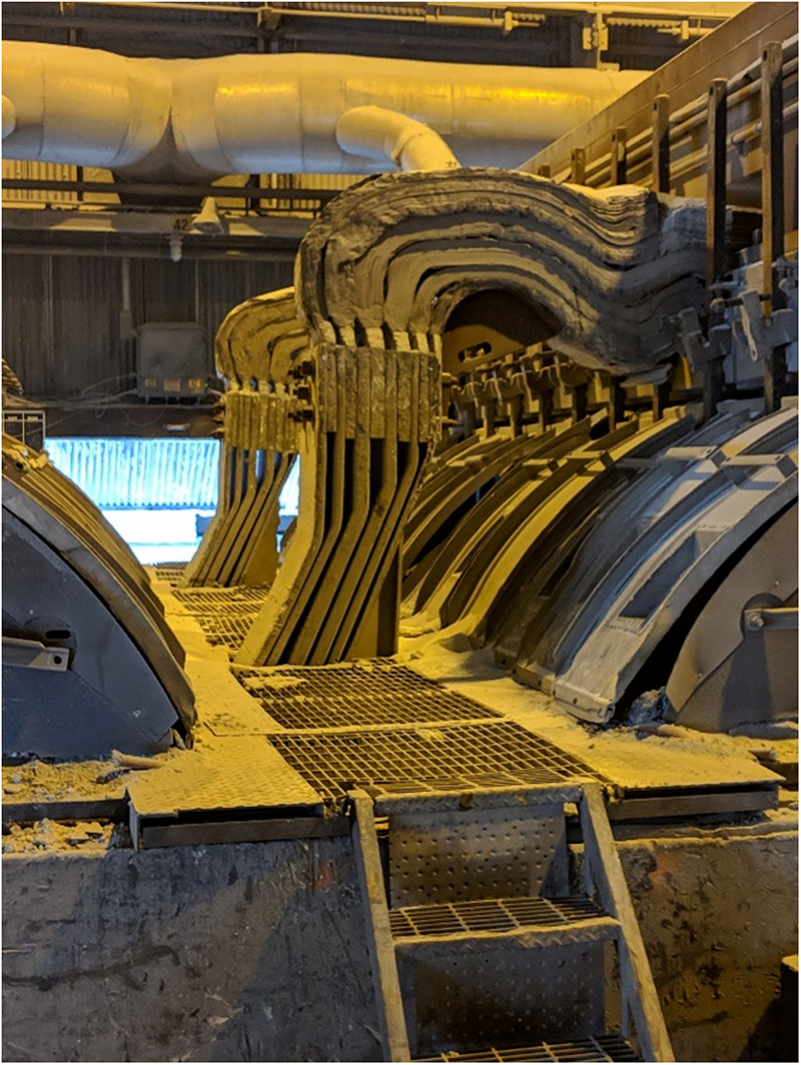
“Catwalk” between two sequential pots showing in‐place covers over electrolytic bath [Color figure can be viewed at wileyonlinelibrary.com]

Within 6 hours of starting his first shift, while performing routine tasks that involved the occasional presence on the “catwalk” between sequential pots (Figure 2), he began to experience hand‐cramping. Shift safety personnel were notified, and the worker was encouraged to access available fluids. The worker continued to perform his assigned job tasks, however, and near the end of his shift at approximately 6.00 AM, he experienced hand‐cramping again, mentioning this to a coworker but not the crew leader or supervisor. He finished his shift and clocked out.

Forty‐five minutes later the site plant protection office received a request to send an ambulance to the shower house for a worker with difficulty breathing. Upon arriving at the scene, the dispatched emergency medical technician (EMT) found the operator walking out of the building and describing body cramps but without concern regarding his breathing. He was noted to be diaphoretic. The worker entered the ambulance under his own power, a presumptive assessment of heat exhaustion was made, and first aid attention consisting of oral fluids and rehydration salts was provided in accordance with site protocol. Initial vital signs showed an oral temperature of only 36.5°C (97.7°F), blood pressure of 160/80 mm Hg, pulse rate of 91 beats per minute, respiratory rate of 18 breaths per minute, and pulse oximetry of 98% (SpO_2_—peripheral oxygen saturation). Recognizing the temperature as unexpectedly low, which was thought secondary to the ingestion of cool rehydration fluids, the EMT intended to repeat this measurement within a few minutes; however, ensuing acute medical events took precedence and a repeat temperature was never obtained in the field.

During transport to the site medical department, the worker experienced a generalized seizure with, as described by the EMT, decorticate posturing, following which he became agitated, combative, and pugnacious. Random blood glucose was 142 mg/dL (7.9 mmol/L). The community ambulance service was summoned, the worker was stabilized with haloperidol and midazolam, and transferred to the proximate regional hospital emergency department for further evaluation and care.

In the emergency department his initial measured oral temperature was 38.1°C (100.6°F), blood pressure 178/89 mm Hg, pulse rate 100 beats per minute, respiratory rate 16 breaths per minute, SpO_2_ 95%, and body mass index (BMI) 34.62 kg/m^2^. Presenting serum sodium level was 114 mmol/L (reference range, 136‐145), with creatinine 1.6 mg/dL (reference range, 0.6‐1.2) and white blood cell count 24.0 thousand/uL (reference range, 4.1‐10.9). Anion gap was calculated at 31 mmol/L (reference range, 7‐14) with serum lactic acid level of 12.4 mmol/L (reference range, 0.5‐2.0). The initial total creatinine phosphokinase (CPK) level was 7641 units/L (reference range, 36‐174). Microscopic urinalysis indicated the presence of a large amount of blood but was otherwise negative. A urinary myoglobin level was not obtained. Acetaminophen and salicylate levels were within normal limits. A seven‐panel urine drug screen was negative for any substances, including amphetamines/methamphetamines or derivatives. Serum alcohol was less than 10 mg/dL (reference range, <10). Arterial blood gas was consistent with metabolic acidosis. The remaining laboratory values upon admission are noted in Table 1. Admitting diagnoses included: hyponatremia (ICD‐10‐CM, E87.1), agitation (R45.1), acute delirium (R41.0), acute respiratory failure (J96.00), acute encephalopathy (G93.40), rhabdomyolysis (M62.82), seizure (G40.89), aspiration (J95.89), and lactic acidosis (E87.2).

**Table 1:** Admission laboratory data

	Value	Reference range
Sodium, mmol/L	114	136‐145
Potassium, mmol/L	3.5	3.5‐5.4
Chloride, mmol/L	72	98‐107
Carbon dioxide, mmol/L	11	20‐33
Creatinine, mg/dL	1.6	0.6‐1.2
Glucose, mg/dL	163	70‐99
White blood cell count, thousands/µL	24	4.1‐10.9
Hemoglobin, g/dL	14.2	12.9‐16.6
Hematocrit, %	41.2	38.0‐48.0
Platelet count, thousands/µL	224	130‐400
Aspartate aminotransferase, units/L	125	0‐39
Alanine aminotransferase, units/L	47	10‐40
Creatinine phosphokinase, units/L	7641	36‐174
Anion gap, mmol/L	31	7‐14
Lactic acid, mmol/L	12.4	0.5‐2.0
Prothrombin time, s	9.8	9.4‐11.4
International normalized ratio	1.0	0.9‐1.1
pH arterial	7.20	7.34‐7.45
Partial pressure carbon dioxide (pCO_2_) arterial, mm Hg	53.4	32‐45
Bicarbonate (HCO_3_) arterial, mm Hg	19.9	20‐26
Base deficit	8.2	0
Fibrinogen, mg/dL	NA	150‐400
Fibrin degradation products, mg/L	NA	<10
D‐dimer, ug/mL FEU	NA	<0.5

Abbreviations: FEU, fibrinogen equivalent units; NA, not available.

Airway protection via intubation was achieved. However, he aspirated during the difficult procedure and remained intubated for the first 48 hours of hospitalization. Day‐of‐admission computerized tomography scan of the head without contrast showed no intracranial mass, hemorrhage, hydrocephalus, or acute intracranial abnormality. An electrocardiogram (EKG) showed sinus tachycardia.

Past medical history was notable for essential hypertension, gastroesophageal reflux disease, and mild attention deficit hyperactivity disorder (ADHD), for which the worker had been prescribed losartan, lansoprazole, and amphetamine/dextroamphetamine, respectively. He was a former smoker and admitted to moderate alcohol ingestion several times per week. He had no known history of prior HRI. However, in the aftermath of this incident, he discovered through family medical history inquiry that he had a history of “cramping” in relation to sports activities and exertion in his youth. There was no known history of hyponatremia or alcohol‐use disorders.^13^, ^14^

Stabilization treatment included rehydration with normal saline solution per recommendation of the consulting nephrologist. An electroencephalogram (EEG) on admission day 2 was indicative of moderate encephalopathy of nonspecific etiology, but without evidence for epileptiform activity. *Enterococcus faecalis* sepsis was diagnosed by blood culture on admission day 4. Hepatitis serologies were nonreactive, as were human immunodeficiency virus 1 and 2.

The worker spent a total of 13 days in the hospital, the first five being in the intensive care unit. Total CPK, aspartate aminotransferase (AST), and alanine aminotransferase (ALT) values in units/L peaked at 125 000, 1478, and 552, respectively, on the fourth hospital day, with a progressive decline in values thereafter (Table 2).

**Table 2:** Chronology of total creatinine phosphokinase (CPK), aspartate aminotransferase (AST), and alanine aminotransferase (ALT) values

	CPK	AST	ALT
Reference range, units/L	36‐174	0‐39	10‐40
Day 1	7641	125	47
Day 2	>22 000	763	193
Day 3	>22 000	1011	305
Day 4	125 850	1478	552
Day 5	79 500	726	499
Day 6	>22 000	519	478
Day 7	18 617	NA	NA
Day 8	11 026	194	387
Day 9	6794	NA	NA
Day 10	4122	NA	NA
Day 11	2758	NA	NA
Day 12	2862	NA	NA
Day 13	2501	NA	NA

Abbreviation: NA, not available.

The hospital discharge summary listed “severe hyponatremia likely triggered by heat exposure” among the discharge diagnoses, with a further notation that the “hyponatremia was likely related to heat stroke.” At the time of discharge, he had pain and weakness in both lower extremities, requiring the use of ankle‐foot orthoses to support standing and walking, as well as numbness and tingling of his upper extremities, primarily his hands.

The worker subsequently underwent a diagnostic evaluation for his apparent polyneuropathy. A noncontrast magnetic resonance imaging (MRI) of the head performed 6 weeks after the incident and 4 weeks postdischarge from the hospital revealed an approximately 1 × 2 cm lobulated nonenhancing cerebellar lesion involving the superior aspect of the left half of the vermis. Repeat noncontrast MRI of the head performed at 20 weeks after the incident revealed stable findings, with no change of this cerebellar lesion.

At 32 weeks after the incident, he remained out of work pending further recovery in his musculoskeletal functional status, resolution or stabilization of the neuropathies, and disposition review regarding return to work in a high‐heat stress environment.

## 3 EXPOSURE ASSESSMENT

Pot operations on the day of the incident were normal. During the worker's evening shift, the average outdoor ambient dry bulb temperature (DBT) was 20.6°C (69°F), with a range of 20°C (68°F) to 22.2°C (72°F). Outdoor humidity during the work shift ranged from 84% to 96%. The average outdoor wet bulb temperature (WBT) calculated from these data was 19°C (66.2°F). The DBT and WBT would have been higher inside the potroom because of process heat, but no data were available from within the potroom. We, therefore, used the average outdoor ambient DBT and WBT and an estimate of globe temperature close to open pots from the literature to estimate the wet bulb globe temperature (WBGT) close to open pots.^11^

When working close to open pots the radiant heat load from molten metal is high. Globe temperatures of up to 188°F (87°C) have been reported.^11^ This means the WBGT, using the aforementioned approach to estimation, could have been as high as 33°C (91.4°F) close to open pots, which exceeds the American Conference of Governmental Industrial Hygienists Threshold Limit Value for acclimatized workers undertaking light work less than 25% of the time.^15^

## 4 DISCUSSION

The PCO job description entails the monitoring and maintenance of routine smelter pot function, the changing of anodes, tapping to remove molten metal, and the operation of an overhead crane to move various raw materials. The electrolytic solution within the pot, called bath, is made up of molten cryolite and aluminum fluoride, maintained at a temperature of around 960°C to 980°C (1760°F to 1796°F). Liquid aluminum is deposited at the bottom of the pot. The hot electrolytic solution within the pot is shielded by a series of aluminum covers, which are periodically manually removed to perform required control tasks (Figure 2). This is when the PCO is most at risk for heat stress.

New to the job, first day back after absence, no or inadequate acclimatization, task inexperience, heavy exertion in the context of elevated outdoor ambient temperature and/or humidity, chronic medical conditions and medications, prior heat‐related illness, poor physical conditioning, alcohol or substance use/abuse, dehydration, and heat‐trapping personal protective equipment (PPE) are all recognized risk factors for HRI.^11^, ^16^ The task of replacing spent carbon anodes, when performed within a smelter that requires significant manual labor at the pot face (manual setting) with the attendant radiant heat load, is an additional notable risk factor for HRI. Enhanced overhead crane‐supported carbon anode replacement helps to lower this risk through a reduction in the proportion of manual tasks required.

The affected employee had worked for the company for less than 5 months, had been away from the workplace for 7 days (returning without a formal re‐acclimatization schedule), was performing manual setting work at a metabolic rate category considered moderate to heavy, had a BMI level that bordered on class II obesity, was under active treatment for chronic hypertension, and had a remote childhood history for sports exertion‐induced heat cramps.

Even when outdoor ambient temperatures are mild, there is the potential for body core temperatures to rise too much and cause exertional heat stroke. This is particularly so if the worker undertakes heavy work, which generates additional metabolic heat, or is exposed for too long before taking a break. Typically, experienced crews are able to pace themselves well enough that severe HRI, most importantly heat stroke, does not occur. They judge how long to stay in front of open pots before taking a break in the aisle and factor into this decision how heavy the task is that they are undertaking. In other words, they can effectively judge work‐rest cycling when working on pots.

The smelter had been restarted 9 months before this incident after a period of curtailment lasting 16 months. Smelter restarts require special care because individual workers, whole crews and even supervisors may not have the experience needed to effectively judge work‐rest cycling when working on pots. In addition, because of inexperience, the completion of manual tasks may take longer, increase metabolic heat and prolong exposures to radiant heat. Also, it is possible that enthusiastic new hires may want to impress and work beyond their abilities—at higher work rates or for longer periods than an experienced worker would choose to do. For the same reason they may choose to ignore symptoms of early HRI, or simply under appreciate their importance as a warning of worse to come.

Comprehensive heat stress management training repeated frequently during the “hot season” and when dictated by specific high‐heat risk tasks or conditions, were in place at the incident location. The relatively low outdoor ambient temperature created a false impression that heat stress risk was low, without adequate recognition of the extreme radiant heat load imparted by working adjacent to open pots. Despite a 7‐day absence from the workplace during the high‐heat season, the worker was not re‐acclimatized as per protocol. Location personnel expressed the general impression that life‐threatening HRI would manifest only in linear fashion after gradual progression from mild HRI, thus the rapid onset of severe symptoms without foretelling observable changes in this worker was unexpected at this location. The encouragement to drink more hydration fluids, once the subclinical physiologic cascade underlying severe HRI had been initiated, may have compounded the hyponatremia that was rapidly evolving through the further dilution of serum sodium levels.

During the acute phase of his hospitalization, differential diagnostic considerations—including acute alcohol withdrawal with delirium tremens, metabolic or toxic encephalopathy (poisoning), CNS infection or encephalitis, thyrotoxicosis, and drug toxicity—were contemplated and ruled out. Although he had been prescribed amphetamine/dextroamphetamine for ADHD, the negative urine drug screen on admission precludes the possibility that amphetamines contributed to his severe HRI.

Exercise‐associated hyponatremia (EAH) and exercise‐associated hyponatremic encephalopathy (EAHE) are additional differential diagnosis considerations. EAH refers to a low blood sodium concentration, typically below 135 mmol/L depending on laboratory, occurring in response to sustained physical exertion, which may present with or without associated symptoms. The most important risk factor for EAH is sustained excessive fluid intake in volumes exceeding sweat, respiratory and renal losses.^17^ EAHE is the more severe life‐threatening variant, occurring when there are concomitant signs or symptoms such as altered mental status, confusion, or seizures.^17^, ^18^, ^19^

EAH is a plausible explanation for the altered mental status and cramping in this case. However, hyponatremia on admission is not unusual in heat stroke and has ranged from 7.4% to 79% of cases in reported series.^20^, ^21^, ^22^, ^23^, ^24^, ^25^, ^26^ In heat stroke, hyponatremia has been attributed to renal sodium loss.^20^

Heat stroke associated systemic inflammatory pathophysiology could have further contributed to CNS dysfunction and encephalopathy.^3^ When the hallmark findings of heat stroke are present, namely, core temperature above 40°C (104°F) and CNS dysfunction, the diagnosis is straightforward. However, there is an overlap in clinical characteristics between heat stroke and EAH/EAHE, which makes definitive diagnosis challenging.

EAH has been repeatedly observed and reported in endurance athletes such as marathon and ultramarathon runners, triathletes, and cyclists and, more recently, other participants in team sports, police officer trainees, and military recruits.^17^, ^18^, ^19^, ^27^ Manual carbon setting in a smelter is an arduous physical job involving exposure to extreme radiant heat indoors, whereas EAH has typically been reported in outdoor settings, which are thermally less stressful. A comprehensive PubMed search returned no case report of EAH in an industrial worker.

The majority of EAH cases occur in individuals who have experienced weight gain, and the risk increases with increasing weight gain.^28^ Hyponatremia has been associated with weight gain—odds ratio 4.2 (95% CI, 2.2‐8.2) for weight gain vs weight loss.^28^

Overhydration, along with physiologically mediated fluid retention, are the principal etiologic factors in EAH. The worker stated that during his work shift he was regularly drinking V8 Vegetable Juice (http://www.campbellsfoodservice.com/brands/v8/) plus other oral rehydration salt drinks. The total volume was estimated at approximately 500 ml/h across the work shift, which is less than the potential maximum compensatory renal loss of 800 to 1000 ml/h.^17^ In addition, given the strenuous job in hot conditions, it is likely that the sweat rate would have been similar to his fluid intake.

Rhabdomyolysis has been reported in association with EAH, with CPK levels typically highest at time of presentation.^29^, ^30^, ^31^, ^32^, ^33^ AST and ALT follow a similar temporal pattern. In exertional heat stroke, AST and ALT usually reach a maximum in about 48 hours.^34^ CPK, AST, and ALT in our case peaked at postincident day 3, favoring heat stroke over EAH, or heat stroke and EAH. AST and ALT values above 1000 units/L within 24 hours have been associated with more frequent hepatic, renal, and cerebral complications and deaths in a series of 35 cases.^34^

The interpretation of core temperature in potential heat stroke cases must be done with caution because temperature can return towards normal before emergency response or medical personnel assessment occurs.^3^, ^5^ On the impression that the initially measured oral temperature of 36.5°C (97.7°F) at the worksite was inaccurate and inconsistent with the overall symptom presentation, the EMT had initiated cooling measures in the field consisting of removal of all upper body clothing, administration of cool oral rehydration fluids, and placing the employee in an air‐conditioned ambulance at 21°C (70°F), where he had been for nearly 10 minutes at the time the seizure occurred. These measures may have further blunted the worker's temperature. No additional cooling measures, such as cold‐water immersion or evaporative cooling, were administered. Oral temperatures underestimate rectal (core) temperatures by an average of 0.5°C.^35^ Delayed hospital‐obtained temperature measurements often also underestimate pre‐hospital temperatures, with an average difference of 3°C reported in one exertional heat stroke series.^5^ Thus, at the time of incident at the smelter, this worker's temperature may have been over 41°C (105.8°F), reflecting the sum of the measured emergency department temperature taken approximately 90 minutes later (38.1°C [100.6°F]) plus the average 0.5°C difference between oral and rectal temperatures, plus the average 3°C difference between pre‐hospital and hospital measurements. While a temperature in this range would have been high enough to corroborate a diagnosis of heat stroke, unfortunately, no accurate temperature was recorded in the field to confirm this possibility.

Had this worker undergone a more formal clinical assessment by the on‐site medical department when his hand cramping first occurred, or reoccurred late into his shift, it is possible that additional signs, symptoms, vital signs, and corroborating laboratory data suggestive of serious HRI may have been identified. In contrast, the symptoms were perceived to be minor and self‐limited.

It is not yet clear what pathology is responsible for the abnormalities seen on MRI of the cerebellum in this case. Importantly, increased MR signal intensity has been reported in the cerebellum of patients in the acute phase of heat stroke, with subsequent scans revealing cerebellar atrophy months later.^36^, ^37^, ^38^ Pathological processes that can result in cerebellar injury during heat stroke include ischemia, hemorrhage, and direct thermal cytotoxicity.^36^, ^37^, ^38^, ^39^ Peripheral neuropathy has been reported before in heat stroke cases.^40^, ^41^ Although there is reference to encephalopathy in EAH, we found no reports of cerebellar pathology or peripheral neuropathy in association with this condition.

In sum, the exposure circumstances and clinical characteristics of this worker present a complex picture of HRI manifestations. The profound hyponatremia strongly aligns with EAH/EAHE as the principal diagnosis. However, hyponatremia has also been reported in heat stroke.^20^, ^21^, ^22^, ^23^, ^24^, ^25^, ^26^ If indeed this worker experienced EAH/EAHE then this would be the first reported case of this entity in an industrial worker. Both EAH/EAHE and heat stroke are sufficient to explain the CNS dysfunction. The pattern of CPK, AST, and ALT changes, and the cerebellar and peripheral nerve abnormalities are more consistent with heat stroke as the primary diagnosis. It is possible that both diagnoses are supported by the totality of the clinical, radiographic, and laboratory findings.

Reinforcement of existing control measures and remediation actions stemming from this incident include the following:

- Evaluation for pot room modernization opportunities to reduce work rate and heat stress load.
- Ensuring adequate crew numbers are maintained to enable sufficient rest periods based on heat stress evaluations.^15^
- Extending heat stress prevention plan focus dates to start before the traditional “hot months” and continuing into the early fall.
- Deploying selective use of preshift urine‐specific gravity testing, particularly for high‐risk tasks and workers.^42^, ^43^
- Increasing contact by line supervisors with workers performing high‐risk tasks.
- Enhancing attention during smelter restarts to the risk of heat illness amongst workers working on open pots, incorporating crew composition that ideally includes an experienced supervisor and one or more experienced crew members, especially in smelters that perform manual carbon setting.
- Short‐term physiological monitoring to assist crew members to learn how to manage their work without excessive exercise heat strain, particularly when experienced supervision or crew composition is lacking.^15^
- Training for workers working on open pots that emphasizes the need for crews to learn to effectively judge work‐rest cycling, specifically addressing the need to not work beyond a person's own limits, and for the symptoms of HRI to trigger immediate evaluation at the site medical center or by emergency medical response personnel.^4^, ^44^
- Rededication to adherence to defined acclimatization protocols during the first week back, for workers who have been absent for a week, with shorter exposures to open pots and shorter periods of heavy tasks.^4^
- Re‐education on proven beneficial hydration practices before, during and after work in high‐heat stress environments, with the aims of replacing fluid lost through sweat, achieving a pale yellow urine color, and preventing a body mass loss of 2% across a shift.^4^, ^42^

## 5 CONCLUSION

Our case describes an abrupt presentation of life‐threatening HRI in an aluminum smelter worker performing traditional pot room tasks. Features of both EAH/EAHE and exertional heat stroke predominated in the clinical evaluation and response to treatment. The job, environmental, clinical, and epidemiological characteristics of this worker strongly parallel those features previously reported to increase the risk for HRI. The importance of radiant heat load as a significant risk for HRI, even in the context of a relatively low outdoor ambient heat index, is highlighted. Reinforcement of known effective heat stress management practices, including predeployment medical evaluation and fitness‐for‐work risk assessment, acclimatization, worker education, rehydration protocols, a focus on crew composition, experience, and work‐rest cycling, and quick assertive reaction to potential evolving HRI in the occupational setting are paramount.

## CONFLICTS OF INTEREST

Dr. Wesdock is the Global Health Director of Alcoa Corporation and Dr. Donoghue is the Global Medical Director of Alcoa Alumina. Both are full‐time employees of Alcoa Corporation and hold shares in the company.

## DISCLOSURE BY AJIM EDITOR OF RECORD

Rodney Ehrlich declares that he has no conflict of interest in the review and publication decision regarding this article.

## AUTHOR CONTRIBUTIONS

JCW initiated the development of this case report, served as liaison to the affected location stakeholders, conducted the emergency response team and medical records review, and acquired the necessary data to prepare the incident timeline chronology and medical laboratory data summary. AMD provided the heat stress assessment analysis and insights and identified the reported associations between heat stroke and cerebellar injury. Both authors contributed to the design and outline of the work, including the supporting literature review and bibliography, and the writing of the manuscript. Both authors gave final approval of the version to be published and agree to be accountable for all aspects of the work in ensuring that questions related to the accuracy or integrity of any part of the work are appropriately investigated and resolved.

## ETHICS APPROVAL AND INFORMED CONSENT

There was no ethics review and approval, however, written informed consent was obtained from the worker described in this case report. Relevant medical records were acquired and maintained in accordance with applicable disclosure laws and practice, and consistent with Alcoa's global data privacy standards.
